# Distribution and Determinants of Antibiotic Self-Medication: A Cross-Sectional Study in Chinese Residents

**DOI:** 10.3390/antibiotics14070701

**Published:** 2025-07-12

**Authors:** Guo Huang, Pu Ge, Mengyun Sui, He Zhu, Sheng Han, Luwen Shi

**Affiliations:** 1 Department of Pharmacy Administration and Clinical Pharmacy, School of Pharmaceutical Sciences, Peking University, Beijing 100191, China; 2 International Research Center for Medicinal Administration, Peking University, Beijing 100191, China; 3 School of Traditional Chinese Medicine, Beijing University of Chinese Medicine, Beijing 100029, China; 4 Division of Chronic Non-Communicable Diseases and Injury Prevention, Shanghai Municipal Center for Disease Control and Prevention, Shanghai 201106, China

**Keywords:** antibiotic self-medication, antimicrobial resistance, determinants, distribution, China

## Abstract

Antimicrobial resistance (AMR) represents a critical global health threat, with inappropriate antibiotic self-medication (ASM) being a key contributor. China—as the world’s largest antibiotic consumer—faces significant challenges despite regulatory efforts, compounded by limited contemporary data during the COVID-19 pandemic. A nationwide cross-sectional study was conducted using the 2021 China Family Health Index Survey (*n* = 11,031 participants across 120 cities). Trained investigators administered face-to-face questionnaires assessing ASM practices, decision-making factors, and sociodemographic characteristics. Multivariate logistic regression identified determinants of ASM. Overall, ASM prevalence was 33.7% (*n* = 3717), with no urban-rural difference (*p* > 0.05). Physician advice (78.2%), drug safety (67.1%), and efficacy (64.2%) were primary selection criteria; rural residents prioritized drug price and salesperson recommendations more than their urban counterparts (*p* < 0.01). Key predictors included higher ASM odds among females (OR = 1.30, 95%CI:1.18–1.43), middle-aged adults (46–59 years; OR = 1.20, 95%CI:1.02–1.42), those with health insurance (resident: OR = 1.33; commercial: OR = 1.62), and individuals with drinking histories (OR = 1.20, 95%CI:1.10–1.31). Lower odds were associated with primary education (OR = 0.69, 95%CI:0.58–0.81), unemployment (OR = 0.88, 95%CI:0.79–0.98), and absence of chronic diseases (OR = 0.56, 95%CI:0.47–0.67). One-third of Chinese residents engaged in ASM during the pandemic, driven by intersecting demographic and behavioral factors. Despite converging urban-rural prevalence rates, distinct decision-making drivers necessitate context-specific interventions, including strengthened pharmacy regulation in rural areas, tailored education programs for high-risk groups, and insurance system reforms to disincentivize self-medication.

## 1. Introduction

Antimicrobial resistance (AMR) constitutes a critical global health threat, imposing substantial economic and human costs, with projections indicating 10 million annual deaths and a 2.5–3% global GDP reduction by 2050 if unaddressed [1,2]. This crisis is acutely pronounced in low- and middle-income countries like China, where antibiotic overuse remains prevalent—exemplified by >80% antibiotic prescription rates for upper respiratory infections and per capita consumption rates fivefold higher than Western nations [3,4]. Inappropriate antibiotic practices, particularly self-medication (ASM)—defined as antibiotic use without professional diagnosis or prescription—accelerate AMR propagation while risking adverse drug reactions, treatment failures, and increased healthcare expenditures [5,6,7].

Global initiatives, including WHO action plans (2011, 2015) and G20 commitments (2016), underscore AMR’s urgency [8,9,10]. China has responded through surveillance systems (e.g., Center for Antibacterial Surveillance, 2005) and policies like China’s National Action Plan to Contain Antimicrobial Resistance (2016–2020) [11,12]. Despite these efforts, non-prescription antibiotic access persists nationwide, with 63–88% of pharmacies dispensing antibiotics without prescriptions [13,14], fueling widespread ASM. Internationally, ASM prevalence varies significantly—from 50.8% in Asian populations to <20% in France and Australia—reflecting disparities in healthcare access, regulatory enforcement, and cultural norms [15,16,17].

Critical knowledge gaps impede effective antibiotic stewardship interventions in China. First, while existing policies primarily target prescribers, patient-centered strategies remain underdeveloped [18]. Second, disparities in healthcare access between rural and urban areas—such as variations in pharmacy proximity versus hospital wait times—exacerbate ASM in underserved regions [19]. China’s rural healthcare system faces structural constraints that influence medication-seeking behaviors. In 2023, the physician-to-population ratio in rural areas (2.74 per 1000 residents) remained significantly lower than in urban centers (4.13 per 1000) in 2023 [20]. Pharmacist distribution is similarly imbalanced; while China’s national average reached 6.0 licensed pharmacists per 10,000 people as of May 2025, the majority remain concentrated in urban areas [21]. Primary care facilities (township health centers/village clinics) often lack essential diagnostic resources and specialist providers, resulting in fragmented care delivery [22]. Consequently, rural residents frequently bypass local facilities for urban hospitals, with studies indicating the majority of patients seek care in cities for serious conditions [23]. These access barriers, compounded by financial constraints and travel burdens, create fertile ground for antibiotic self-medication as a pragmatic alternative. Third, although the COVID-19 pandemic has altered antibiotic usage patterns, population-level data during this period are scarce [24]. Finally, behavioral determinants—including substance use and socioeconomic factors—are not comprehensively assessed despite their documented influence on health decision-making [25].

This study addresses these gaps through a national survey of Chinese residents in 2021, aiming to quantify ASM distribution post-COVID-19 emergence, identify key considerations driving antibiotic procurement, and analyze sociodemographic, behavioral, and regional determinants of ASM, thereby informing targeted antimicrobial stewardship strategies aligned with Healthy China 2030 goals.

## 2. Results

### 2.1. Study Participants

The study enrolled 11,031 eligible participants, comprising 8008 (72.60%) urban and 3023 (27.40%) rural residents. Over half were female, with nearly half aged ≤30 years. A normal BMI range was observed in 68.40% of participants, while more than half were married, highly educated, and unemployed. Most (70.57%) reported a monthly household income per capita exceeding CNY 3000 (USD 420), and 17.63% had ≥1 chronic disease. Non-smokers and non-drinkers constituted 19.82% and 40.37% of the cohort, respectively.

Compared to urban residents, those in rural areas exhibited significantly higher proportions of individuals aged ≥60 years, without spouses, unemployed, with monthly household income per capita < CNY 3000, covered by out-of-pocket payment medical insurance, and without alcohol consumption history, but lower proportions of higher education attainment, absence of chronic diseases, and no smoking history (Table 1).

### 2.2. Distribution of ASM

Among 11,031 participants, 3717 (33.70%) reported practicing ASM. ASM prevalence varied significantly across sociodemographic strata (Table 2), with higher rates observed among females, middle-aged adults (46–59 years), overweight individuals, married participants, highly educated respondents, white-collar workers, urban residents, and those with chronic diseases, smoking history, or drinking history. Lower ASM rates were associated with out-of-pocket medical insurance and the absence of chronic diseases. No significant differences existed for income.

### 2.3. Considerations of ASM Practitioners

**Figure 1:**
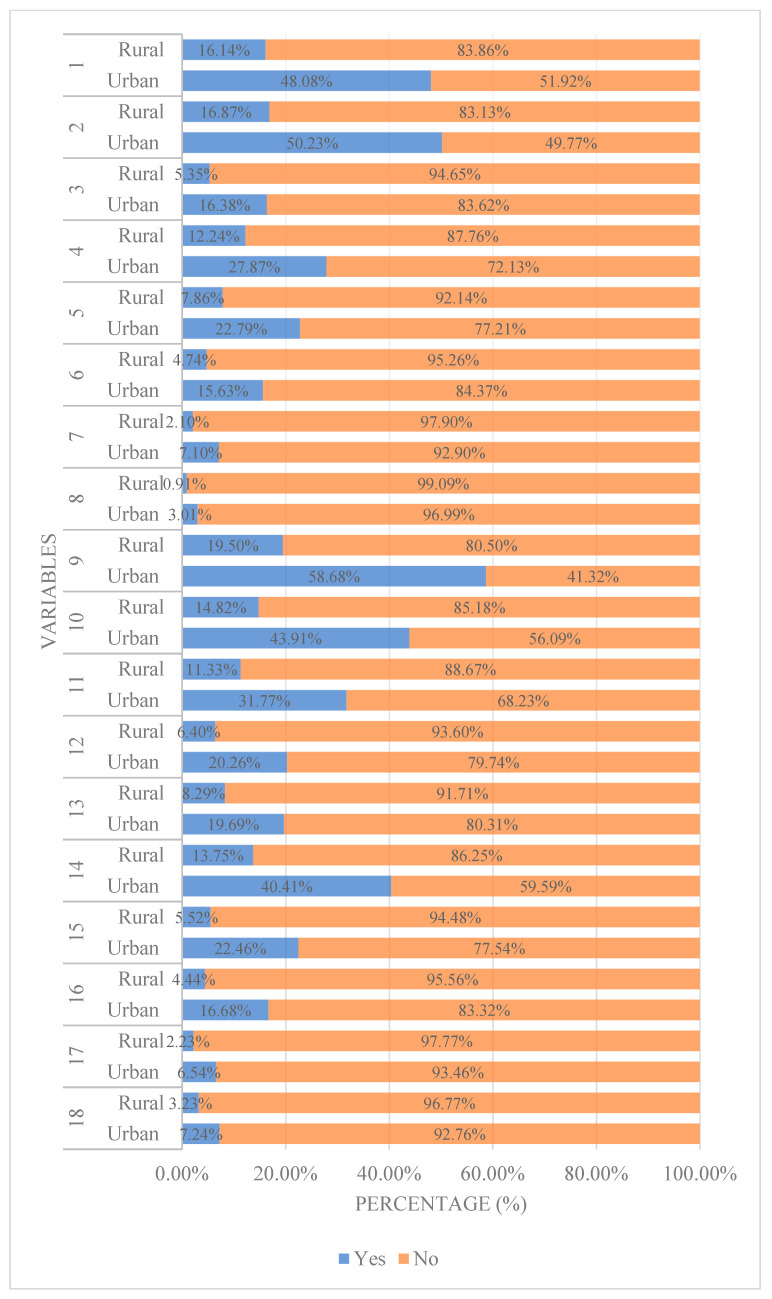
Considerations distribution of ASM Practitioners.

The 3717 ASM practitioners prioritized three key factors when selecting antibiotics: physicians’ advice (78.18%), drug safety (67.10%), and drug efficacy (64.22%) (Table 3; Figure 1). Rural residents placed significantly greater emphasis on drug price, recommendations from sales personnel, and after-sales service, whereas urban residents valued brand reputation and corporate credibility more highly.

### 2.4. Determinants of ASM

Multivariate logistic regression identified several independent predictors of ASM (Table 4). Males exhibited 23% lower odds of ASM than females (OR = 0.770; 95% CI: 0.700–0.845; *p* < 0.0001). Middle-aged adults (46–59 years) had 20.3% higher odds than elderly participants (≥60 years) (OR = 1.203; 95% CI: 1.020–1.418; *p* = 0.0279). Those with primary/below education showed 31.3% lower odds versus higher-educated counterparts (OR = 0.687; 95% CI: 0.580–0.813; *p* < 0.0001). Unemployed participants had 12.1% lower odds relative to white-collar workers (OR = 0.879; 95% CI: 0.788–0.981; *p* = 0.0209). Participants with resident/employee health insurance (OR = 1.327; 95% CI: 1.191–1.478; *p* < 0.0001) or commercial insurance (OR = 1.624; 95% CI: 1.227–2.149; *p* = 0.0007) demonstrated higher odds than those relying on out-of-pocket payment. Absence of chronic diseases predicted lower odds (none vs. multiple: OR = 0.561; 95% CI: 0.469–0.671; *p* < 0.0001; one vs. multiple: OR = 0.715; 95% CI: 0.588–0.869; *p* = 0.0008). Drinking history increased ASM odds by 20.1% (OR = 1.201; 95% CI: 1.097–1.314; *p* < 0.0001).

## 3. Discussion

This nationwide study reveals a 33.7% prevalence of ASM among Chinese residents during the COVID-19 pandemic. Notably, despite significant urban-rural socioeconomic disparities, no difference in ASM prevalence was observed, suggesting this practice remains deeply embedded across diverse communities. This finding highlights the urgent need for context-specific interventions to address AMR, a critical global health threat projected to cause 4.73 million annual deaths in Asia by 2050 that imposes substantial economic burdens [2].

China’s ASM rate aligns with previous national studies reporting 37.1–45.7% prevalence [26,27], but exceeds rates in high-income nations like the UK (5%) and France (18%) [17,28]. While lower than India (58%) [29], it surpasses the WHO’s recommended threshold of 30% [30]. This intermediate position reflects China’s unique healthcare landscape where rapid economic development coexists with persistent self-medication traditions. The convergence of urban and rural ASM rates underscores how cultural norms and medication accessibility may transcend geographic and socioeconomic boundaries [31,32], particularly during healthcare disruptions like the COVID-19 pandemic which affected medication dispensing patterns [33].

Our analysis identified several demographic predictors of ASM. Females exhibited 30% higher odds than males (OR = 1.299), consistent with studies from Sudan and the UK [33,34]. This gender disparity may stem from differential care-seeking behaviors, higher antibiotic prescription rates for women, and greater access to leftover medications [35]. Middle-aged adults (46–59 years) showed elevated risk, potentially due to time constraints and healthcare access barriers [36]. The observed association between higher educational attainment and increased ASM (OR = 1.456 vs. primary education) reveals a critical education paradox: advanced education confers no protective effect on appropriate antibiotic use. This counterintuitive pattern—replicated in Italy [34] and Sudan [37]—suggests knowledge acquisition alone is insufficient for behavior modification. Rather, the findings point to diagnostic overconfidence among educated individuals [38], wherein perceived competence in self-assessment outweighs evidence-based risk awareness. This overconfidence manifests as unwarranted trust in personal judgment regarding antibiotic indication and dosing, inadvertently amplifying AMR risks.

Participants with health insurance demonstrated significantly higher ASM odds (Resident/Employee: OR = 1.327; Commercial: OR = 1.624), aligning with Iranian evidence that insured individuals perceive self-medication as financially lower-risk [39]. Multiple chronic diseases amplified ASM risk (OR = 1.782 vs. disease-free), likely due to symptom familiarity and frequent medication exposure [40], while drinking history increased odds by 20.1% (OR = 1.201), corroborating European associations between substance use and self-medication [41].

While our study identifies critical factors influencing antibiotic selection decisions (e.g., physicians’ advice, safety perceptions), these observations lack explicit theoretical contextualization. Grounding findings in the Beliefs about Medicines Questionnaire (BMQ) framework—which evaluates individuals’ necessity beliefs (perceived need for treatment) and concern beliefs (worries about risks, side effects, or dependence)—provides a structured lens to interpret behavioral drivers [42]. For instance, public reliance on physician advice reflects heightened necessity beliefs, where antibiotics are perceived as essential for symptom resolution. Conversely, safety considerations align directly with BMQ’s concern beliefs, encompassing fears of adverse effects or long-term harm from inappropriate use. Notable urban-rural divergences emerged in antibiotic selection criteria. Rural residents prioritized drug price, salesperson recommendations, and after-sales service, whereas urban consumers emphasized brand reputation and corporate credibility. These differences likely reflect rural healthcare access barriers, medication literacy gaps, and targeted marketing strategies by community pharmacies [43,44]. The substantial rural reliance on salesperson advice is particularly concerning given frequent deficiencies in formal medical training among pharmacy staff.

To curb irrational antibiotic use, collaborative engagement among the public, physicians, and policymakers is imperative. Furthermore, current study findings necessitate implementing more precise, multifaceted interventions.

Fundamental to curbing ASM is the rigorous enforcement of prescription-only dispensing policies, particularly targeting rural pharmacies where non-prescription access persists despite nationwide prohibitions [45]. This necessitates implementing real-time e-prescription verification systems with blockchain auditing to prevent circumvention, coupled with substantial penalties for violations. Concurrently, mandatory pharmacist certification programs must integrate AMR stewardship training [46,47], specifically addressing commercial pressures that drive inappropriate sales in competitive markets [48]. To disrupt the supply reservoir for self-medication, precise tablet dispensing aligned with treatment duration [49,50] should be mandated nationwide, supplemented by community medication take-back networks utilizing existing pharmacy distribution channels.

Public education campaigns must transcend generic awareness to address population-specific misconceptions. Urban interventions should leverage brand psychology by collaborating with reputable manufacturers to embed stewardship messaging in packaging (e.g., “This trusted brand protects your family through evidence-based use”), while deploying WeChat-based “AMR Risk Calculators” that gamify antibiotic literacy [51]. For rural populations, community radio dramas illustrating AMR consequences and IVR prescription reminders can counter salesperson influence [52]. Critically, campaigns must explicitly target educated demographics whose knowledge-practice disconnect reflects diagnostic overconfidence—reframing WHO’s World Antibiotic Awareness Week materials to challenge misconceptions of antibiotics as “panaceas” [53] through cognitive dissonance strategies.

Insurance system reforms should introduce tiered co-payment structures that impose disincentives for non-guideline-compliant antibiotic prescriptions while ensuring full coverage for WHO Essential Medicines List antibiotics [54]. Urban schemes could incorporate prescription behavior monitoring into physician reimbursement algorithms, whereas rural initiatives require subsidized narrow-spectrum antibiotics to alleviate price-driven compromises. China should further capitalize on its telemedicine infrastructure through integrated digital health platforms: WeChat health portals could deliver BMQ-informed patient education modules for urban users, while AI-powered diagnostic support tools on telemedicine platforms enhance prescription accuracy in remote clinics, reducing unnecessary antibiotic demand [55].

Finally, as trusted health authorities, healthcare providers are pivotal in correcting public misconceptions about antibiotic use. Physicians and pharmacists must actively manage patient expectations through evidence-based communication strategies, particularly when counseling high-risk groups identified in this study (e.g., insured females 46–59) [56]. Crucially, providers should lead by example: reducing non-evidence-based antibiotic prescriptions—a persistent issue in primary care—directly challenges the false perception of antibiotics as universally effective remedies. Sustainable change requires systematic implementation and reinforcement of standardized clinical guidelines for rational antibiotic use across all practice settings [57,58].

Several methodological limitations warrant acknowledgment. First, the cross-sectional design precludes causal inference regarding ASM determinants. Second, self-reported data introduces potential recall and social desirability biases. Third, specific antibiotic names and spectral classifications (e.g., broad- vs. narrow-spectrum) were not captured in the survey instrument, restricting our ability to assess differential AMR risks associated with antibiotic types. Fourth, regional specificity limits generalizability beyond China. Finally, the exclusion of healthcare provider perspectives omits critical stakeholders in antibiotic dispensing. Future research should employ longitudinal designs to track ASM evolution post-pandemic, incorporate provider insights, and integrate the WHO AWaRe classification for quantifying spectrum-specific antibiotic exposure, with particular focus on identified high-risk groups such as rural residents prioritizing affordability.

## 4. Materials and Methods

### 4.1. Study Design and Population

Data were derived from the 2021 China Family Health Index Survey (CFHI-2021) [59], conducted from 10 July to 15 September 2021, across 120 cities in 22 provinces and 5 autonomous regions. Trained investigators in each city administered questionnaires via the online Questionnaire Star platform (https://www.wjx.cn/ (assess on 10 July 2021)), using a one-on-one, face-to-face approach. After investigators entered unique questionnaire IDs, respondents independently completed the survey by accessing the provided link. For participants with cognitive capacity but physical limitations, investigators recorded responses based on verbal input. The inclusion criteria were as follows: (1) Age ≥16 years; (2) Response to the ASM question; (3) Provision of written informed consent.

### 4.2. The Questionnaire and Data Collection

The structured questionnaire comprised two sections: 1. Sociodemographic and clinical characteristics: Gender, age, BMI, marital status, education, occupation, monthly household income per capita, medical insurance, chronic disease count, smoking/drinking history, and residence (urban/rural). Urban/rural residency was classified based on China’s household registration (hukou) system. Participants reporting non-agricultural or urban resident hukou status were categorized as urban residents. Those reporting agricultural hukou status were categorized as rural residents. This approach aligns with established socioeconomic stratification in China [60]. Notably, 2. antibiotic self-medication behavior: Question 1 (single-response): “Have you ever purchased and used antibiotics without a prescription?” (Yes/No). Question 2 (multiple-response, for ASM practitioners only): “What factors influenced your antibiotic purchase decisions?” Participants selected from 18 options: (1) Drug efficacy; (2) Drug safety; (3) Dosage form (e.g., capsules, patches); (4) Drug price; (5) Insurance reimbursement eligibility; (6) Ease of administration; (7) Taste of medication; (8) Packaging aesthetics; (9) Physicians’ advice; (10) Pharmacists’ advice; (11) Family members’ suggestions; (12) Friends’ suggestions; (13) Recommendations from sales personnel; (14) Personal experience; (15) Brand reputation; (16) Corporate credibility; (17) Advertising influence; and (18) After-sales service. Respondents selected 1–18 options.

### 4.3. Statistical Analysis

Descriptive statistics summarized categorical variables as frequencies (%) and normally distributed continuous variables as mean ± standard deviation. Chi-square tests identified potential determinants of ASM and urban-rural differences. Binary logistic regression assessed associations between ASM (dependent variable) and sociodemographic factors (independent variables), reporting adjusted odds ratios (adjusted ORs) with 95% confidence intervals (CIs). Statistical significance was set at *p* < 0.05. Analyses used SAS 9.4 (SAS Institute, Cary, NC, USA).

## 5. Conclusions

This study reveals persistent antibiotic ASM practices across both urban and rural China, mediated through factors including gender, education level, insurance status, and health-related behaviors. The convergence of ASM prevalence despite divergent regional socioeconomic profiles necessitates multifaceted interventions. Future research should employ longitudinal designs to track post-pandemic ASM trends and incorporate healthcare provider perspectives. Immediate action is imperative to integrate pharmacist education, public awareness campaigns, and insurance reforms within China’s evolving primary healthcare framework to mitigate the escalating AMR crisis.

## Figures and Tables

**Table 1:** General characteristics of Chinese residents.

Variables	*n* (%)			χ2	*p*-Value
	Urban	Rural	Total		
Total	8008 (72.60)	3023 (27.40)	11,031 (100.00)		
Gender				0.0831	0.7731
Male	3647 (45.54)	1386 (45.86)	5033 (45.63)		
Female	4361 (54.56)	1637 (54.15)	5998 (54.37)		
Age (years)				179.8654	<0.0001
0–30	3377 (42.17)	1288 (42.61)	4665 (42.29)		
31–45	2325 (29.03)	676 (22.36)	3001 (27.21)		
46–59	1652 (20.63)	566 (18.72)	2218 (20.11)		
60–	654 (8.17)	493 (16.31)	1147 (10.40)		
BMI (kg/m^2^)				3.7998	0.1496
<18.5	1103 (13.77)	459 (15.18)	1562 (14.16)		
18.5–24.9	5510 (68.81)	2035 (67.32)	7545 (68.40)		
25–	1395 (17.42)	529 (17.50)	1924 (17.44)		
Spouse				34.7433	<0.0001
Yes	2243 (60.34)	3983 (54.56)	6226 (56.44)		
No	1474 (39.66)	3331 (45.54)	4805 (43.56)		
Education level				832.9165	<0.0001
Primary or below	473 (5.91)	654 (21.63)	1127 (10.22)		
Secondary	2272 (28.37)	1145 (37.88)	3417 (30.98)		
Higher	5263 (65.72)	1224 (40.49)	6487 (58.81)		
Occupation				527.0434	<0.0001
Unemployed	4496 (56.14)	2379 (78.70)	6875 (62.32)		
Blue-collar	992 (12.39)	295 (9.76)	1287 (11.67)		
White-collar	2520 (31.47)	349 (11.54)	2869 (26.01)		
Monthly household income per capita				1020.805	<0.0001
0–3000	1714 (21.40)	1532 (50.68)	3246 (29.43)		
3001–6000	3229 (40.32)	1025 (33.91)	4254 (38.56)		
6001–	3065 (38.27)	466 (15.42)	3531 (32.01)		
Medical insurance				60.1299	<0.0001
Resident/employee	6083 (75.96)	2206 (72.97)	8289 (75.14)		
Commercial	203 (2.53)	34 (1.12)	237 (2.15)		
Government-funded	168 (2.10)	38 (1.26)	206 (1.87)		
Out-of-pocket payment	1554 (19.41)	745 (24.64)	2299 (20.84)		
Number of chronic diseases				11.7185	0.0029
none	6644 (82.97)	2442 (80.78)	9086 (82.37)		
Single	932 (11.64)	369 (12.21)	1301 (11.79)		
Multiple	432 (5.39)	212 (7.01)	644 (5.84)		
Smoking history				15.2551	<0.0001
Yes	1514 (18.91)	672 (22.23)	2186 (19.82)		
No	6494 (81.09)	2351 (77.77)	8845 (80.18)		
Drinking history				42.7765	<0.0001
Yes	3383 (42.25)	1070 (35.40)	4453 (40.37)		
No	4652 (57.75)	1953 (64.60)	6578 (59.63)		

**Table 2:** Distributions of ASM among Chinese residents.

Variables	ASM [*n* (%)]			χ2	*p*-Value
	Yes	No	Total		
Total	3717 (33.70)	7314 (66.30)	11,031 (100.00)		
Gender				6.6819	0.0097
Male	1632 (43.91)	3401 (46.50)	5033 (45.63)		
Female	2085 (56.09)	3913 (53.50)	5998 (54.37)		
Age (years)				55.2949	<0.0001
0–30	1423 (38.28)	3242 (44.33)	4665 (42.29)		
31–45	1020 (27.44)	1981 (27.09)	3001 (27.21)		
46–59	876 (23.57)	1342 (18.35)	2218 (20.11)		
60–	398 (10.71)	749 (10.24)	1147 (10.40)		
BMI (kg/m^2^)				14.5356	0.0007
<18.5	471 (12.67)	1091 (14.92)	1562 (14.16)		
18.5–24.9	2548 (68.55)	4997 (68.32)	7545 (68.40)		
25–	698 (18.78)	1226 (16.72)	1924 (17.44)		
Spouse				34.7433	<0.0001
Yes	2243 (60.34)	3983 (54.46)	6226 (56.44)		
No	1474 (39.66)	3331 (45.54)	4805 (43.56)		
Education level					
Primary or below	324 (8.72)	803 (10.98)	1127 (10.22)	14.7739	0.0006
Secondary	1148 (30.89)	2269 (31.02)	3417 (30.98)		
Higher	2245 (60.40)	4242 (58.00)	6487 (58.81)		
Occupation				48.6309	<0.0001
Unemployed	2156 (58.00)	4719 (64.52)	6875 (62.32)		
Blue-collar	455 (12.24)	832 (11.38)	1287 (11.67)		
White-collar	1106 (29.76)	1763 (24.10)	2869 (26.01)		
Monthly household income per capita				4.7330	0.0938
0–3000	1045 (28.11)	2201 (30.09)	3246 (29.43)		
3001–6000	1454 (39.12)	2800 (38.28)	4254 (38.56)		
6001–	1218 (32.77)	2313 (31.62)	3531 (32.01)		
Medical insurance				60.5866	<0.0001
Resident/employee	2931 (78.85)	5358 (72.98)	8289 (75.14)		
Commercial	95 (2.56)	142 (1.94)	237 (2.15)		
Government-funded	70 (1.88)	136 (1.86)	206 (1.87)		
Out-of-pocket payment	621 (16.71)	1678 (22.94)	2299 (20.84)		
Number of chronic diseases				65.5118	<0.0001
none	2921 (78.58)	6165 (84.29)	9086 (82.37)		
Single	501 (13.48)	800 (10.94)	1301 (11.79)		
Multiple	295 (7.94)	349 (4.77)	644 (5.84)		
Smoking history				6.7482	0.0094
Yes	788 (21.20)	1398 (18.99)	2186 (19.82)		
No	2929 (78.80)	5916 (80.89)	8845 (80.18)		
Drinking history				19.8502	<0.0001
Yes	1609 (43.29)	2844 (38.88)	4453 (40.37)		
No	2108 (56.71)	4470 (61.12)	6578 (59.63)		
Residence				11.0567	0.0009
Urban	2772 (74.58)	5236 (71.59)	8008 (72.60)		
Rural	945 (25.42)	2078 (28.41)	3023 (27.40)		

**Table 3:** Considerations of ASM Practitioners by residence.

Variables	*n* (%)			χ2	*p*-Value
	Urban	Rural	Total		
Total	2772 (74.58)	945 (25.42)	3717 (100.00)		
Clinical factors					
1 Drug efficacy	1787 (64.47)	600 (63.49)	2387 (64.22)	0.2910	0.5896
2 Drug safety	1867 (67.35)	627 (66.35)	2494 (67.10)	0.3211	0.5710
3 Dosage form (e.g., capsules, patches)	609 (21.97)	199 (21.06)	808 (21.74)	0.3442	0.5574
Economic and accessibility					
4 Drug price	1036 (37.37)	455 (48.15)	1491 (40.11)	34.0566	<0.0001
5 Insurance reimbursement eligibility	847 (30.56)	292 (30.90)	1139 (30.64)	0.0392	0.8430
Convenience and experience					
6 Ease of administration	581 (20.96)	176 (18.62)	757 (20.37)	2.3697	0.1237
7 Taste of medication	264 (9.52)	78 (8.25)	342 (9.20)	1.3602	0.2435
8 Packaging aesthetics	112 (4.04)	34 (3.60)	146 (3.93)	0.3657	0.5453
Social and personal advice					
9 Physicians’ advice	2181 (78.68)	725 (76.72)	2906 (78.18)	1.5873	0.2077
10 Pharmacists’ advice	1632 (58.87)	551 (58.31)	2183 (58.73)	0.0937	0.7596
11 Family members’ suggestions	1181 (42.60)	421 (44.55)	1602 (43.10)	1.0879	0.2969
12 Friends’ suggestions	753 (27.16)	238 (25.19)	991 (26.66)	1.4120	0.2347
13 Recommendations from sales personnel	732 (26.41)	308 (32.59)	1040 (27.98)	13.3816	0.0003
14 Personal experience	1502 (54.18)	511 (54.07)	2013 (54.16)	0.0035	0.9530
Brand and corporate					
15 Brand reputation	835 (30.12)	205 (21.69)	1040 (27.98)	24.8509	<0.0001
16 Corporate credibility	620 (22.37)	165 (17.46)	785 (21.12)	10.1830	0.0014
17 Advertising influence	243 (8.77)	83 (8.78)	326 (8.77)	0.0002	0.9874
18 After-sales service	269 (9.70)	120 (12.70)	389 (10.47)	6.7430	0.0094

**Table 4:** Multivariate logistic regression on ASM among Chinese residents.

Variables	β	SE	Wald χ^2^	*p*-Value	OR (95%CI)
Intercept	−0.2714	0.1474	3.3924	0.0655	
Gender (Ref: Female)					
Male	−0.2619	0.0480	29.7837	<0.0001	0.770 (0.700, 0.845)
Age (Ref: 60–)					
0–30	−0.0321	0.0953	0.1133	0.7364	0.968 (0.803, 1.167)
31–45	−0.0108	0.0857	0.0159	0.8997	0.989 (0.836, 1.170)
46–59	0.1848	0.0841	4.8313	0.0279	1.203 (1.020, 1.418)
BMI (Ref: 25–)					
<18.5	−0.1350	0.0771	3.0688	0.0798	0.874 (0.751, 1.016)
18.5–24.9	−0.0362	0.0553	0.4279	0.5130	0.964 (0.865, 1.075)
Spouse (Ref: No)					
Yes	0.0408	0.0610	0.4482	0.5032	1.042 (0.924, 1.174)
Education level (Ref: Higher)					
Primary or below	−0.3759	0.0863	18.9833	<0.0001	0.687 (0.580, 0.813)
Secondary	−0.0769	0.0509	2.2792	0.1311	0.926 (0.838, 1.023)
Occupation (Ref: White-collar)					
Unemployed	−0.1291	0.0559	5.3329	0.0209	0.879 (0.788, 0.981)
Blue-collar	−0.0905	0.0729	1.5406	0.2145	0.913 (0.792, 1.054)
Monthly household income per capita (Ref: 6001–)					
0–3000	0.0330	0.0570	0.3365	0.5618	1.034 (0.924, 1.156)
3001–6000	0.0203	0.0492	0.1707	0.6795	1.021 (0.927, 1.124)
Medical insurance (Ref: Out-of-pocket payment)					
Resident/employee	0.2826	0.0552	26.2324	<0.0001	1.327 (1.191, 1.478)
Commercial	0.4848	0.1430	11.4930	0.0007	1.624 (1.227, 2.149)
Government-funded	0.2163	0.1572	1.8926	0.1689	1.241 (0.912, 1.690)
Number of chronic diseases (Ref: Multiple)					
None	−0.5776	0.0913	39.9822	<0.0001	0.561 (0.469, 0.671)
Single	−0.3353	0.0997	11.3167	0.0008	0.715 (0.588, 0.869)
Smoking history (Ref: No)					
Yes	0.0849	0.0608	1.9542	0.1621	1.089 (0.966, 1.226)
Drinking history (Ref: No)					
Yes	0.1830	0.0461	15.7526	<0.0001	1.201 (1.097, 1.314)
Residence (Ref: Rural)					
Urban	0.0454	0.0501	0.8211	0.3648	1.046 (0.949, 1.154)

## Data Availability

The data presented in this study can be made available upon request from the corresponding author.
